# Supraventricular Tachycardia and Tricuspid Regurgitation in the Setting of Misplaced Implantable Port Catheter Tip

**DOI:** 10.7759/cureus.1460

**Published:** 2017-07-11

**Authors:** Ahmad Awan, Bisma Ahsan, Hasan Iftikhar, Akbar Khan, Fasil Tiruneh, Yididia Bekele, Ankit Mahajan, Ahmed A Awan

**Affiliations:** 1 Department of Internal Medicine, Howard University Hospital; 2 Department of Infectious Disease, Howard University Hospital; 3 Health Information Management, Howard University Hospital; 4 Patch, Irhythm Technologies Inc; 5 Cardiology, Howard University Hospital; 6 Internal Medicine, Forrest General Hospital, Hattiesburg Ms

**Keywords:** tricuspid regurgitation, port-a-cath, transesophageal echocardiogram, supraventricular tachycardia

## Abstract

We present the case of a 31-year-old female with a past medical history of B-cell leukemia, on maintenance chemotherapy administered via centrally placed implantable catheter port, who presented to the emergency room with fever, chills, and generalized body pain of one day's duration. After initial workup, the patient was admitted to the intensive care unit and managed for severe sepsis. The patient was found to have a new-onset 3/6 holosystolic murmur at the left lower sternal border. Furthermore, she developed an episode of supraventricular tachycardia that responded to adenosine. Transthoracic echocardiogram revealed severe tricuspid regurgitation but without valvular vegetation. Transesophageal echocardiogram further confirmed the absence of vegetation, in addition to visualizing the tip of the catheter tip in the right atrium and interfering with coaptation of the tricuspid valve. It was postulated that the severe tricuspid regurgitation and supraventricular tachycardia were caused by the catheter tip malposition. The catheter was subsequently removed. The patient’s acute condition resolved and she was referred to cardiothoracic surgery for valvular surgery.

## Introduction

Port-A-Cath® (Smiths Medical, Dublin, OH) is a propriety central venous access port device (CVAPD), which is the most common modality used for administering chemotherapy in patients with a malignancy. Usually introduced via the jugular vein by an interventional radiologist, the Port-A-Cath is placed by way of a guidewire into the superior vena cava and then tunneled in the chest wall. Post-procedure complications include infection, venous thrombosis, pneumothorax, arrhythmia, and cardiac arrest. Cardiac complications, in particular, can happen when the guidewire comes into contact with right atrium or atrioventricular node (AV node). We report a case of tricuspid regurgitation and supraventricular tachycardia in the setting of a catheter tip malposition thought to be due to the catheter traversing the tricuspid valve each time valve leaflets approximate. 

## Case presentation

A 31-year-old female with a history of B-cell leukemia presented to our emergency room with generalized body pain, fever, and chills of one day's duration. She was diagnosed with B-cell leukemia two years prior and was on a maintenance regimen of vincristine, methotrexate, and 6-mercaptopurine, being administered through Port-A-Cath central venous access port device. This port was placed one year ago without any complications. At the current presentation, she denied having any chest pain, dyspnea, or dizziness. She did not report any focal symptom on a review of her system. Her medications included a chemotherapy regimen and hydromorphone as needed for pain. Her past medical history was otherwise noncontributory. She had not had any recent surgery. She denied tobacco use, alcohol consumption, or using intravenous drugs.

In the emergency room, the patient was febrile (temperature: 102.6^o^F) and tachycardic (heart rate: 120 beats per minute). The patient was normotensive and was saturating 100% on room air. A significant physical exam finding was a 2/6 holosystolic murmur at the left lower sternal border. The subcutaneous port pocket was non-tender and non-erythematous. She also had significant splenomegaly. Neurologic exam was unremarkable with no focal defect discerned.

Labs revealed plasma lactate level of 5.5 mg/dl, a leukocyte count of 3.1 X 10^9 ^per liter, platelet count of 34,000 X10^9^ per liter, and a hemoglobin of 8.5 mg/dl. The patient had an anion gap of 15 and a bilirubin level of 1.4 mg/dl. Initial electrocardiography (EKG) revealed sinus tachycardia. A chest X-ray showed no active parenchymal disease, whereas computer tomography (CT) of the chest, abdomen, and pelvis was unremarkable for acute pathology, except for focal sigmoid colon wall thickening without surrounding inflammatory changes. Of note, the Port-A-Cath tip was terminating in the right atrium. Immediate fluid resuscitation was instituted and cultures were obtained. An intravenous antimicrobial regimen was initiated, and the patient was admitted to the medical ICU.

Subsequently, patient’s heart rate increased to more than 160 beats per minute. Telemetry revealed a supraventricular tachycardia. Carotid sinus massage and Valsalva maneuver did not improve heart rate, and the patient received three doses of adenosine. She was then administered amiodarone, 300 mg intravenous (IV) bolus followed by continuous infusion, which led to an abatement of the tachyarrhythmia.

Her initial course was complicated by worsening anemia and neutropenia. Initial blood cultures returned positive for gram-negative rods in two specimens. A transthoracic echocardiogram revealed new onset tricuspid regurgitation with a mildly elevated right ventricular systolic pressure of 37 mm.

**Figure 1:**
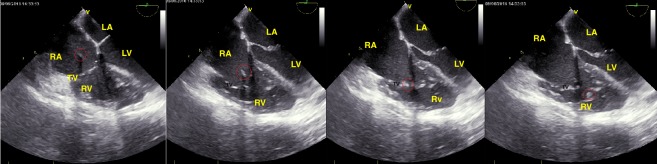
Serial echocardiographic pictures of catheter tip through the cardiac cycle The catheter tip is circled red. TV: tricuspid valve; RV: right ventricle; LV: left ventricle; TV: tricuspid valve; RA: right atrium; LA: left atrium

**Figure 2:**
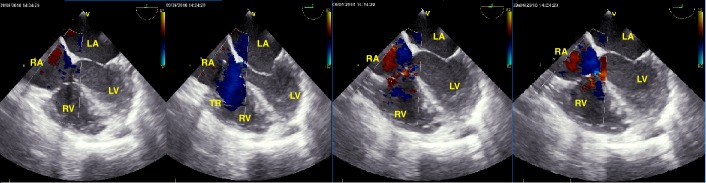
Four-chamber echocardiographic view showing regurgitant jet across the tricuspid valve Blue color signifies regurgitation. TR: tricuspid regurgitation; TV: tricuspid valve; RV: right ventricle; LV: left ventricle; TV: tricuspid valve; RA: right atrium; LA: left atrium

Given the clinical suspicion of endocarditis, a transesophageal echocardiography was performed. The procedure showed no vegetations on the valve surface but did visualize the catheter tip traversing through the right atrial cavity up to the right atrial junction and tricuspid valve, at which point, it was seen to intermittently prolapse through the tricuspid valve (Figure 1). This finding was accompanied by severe tricuspid regurgitation (Figure 2).

It was inferred that the tricuspid regurgitation and supraventricular tachycardia were caused by catheter tip causing mechanical disruption to the tricuspid valve structure. The catheter and port were removed and the catheter tip was cultured. Follow-up microbiology identified blood culture positivity for Escherichia coli, while the catheter tip culture resulted in no growth

The patient was continued on antimicrobial therapy for febrile neutropenia. Her sepsis resolved and she did not develop any further episodes of tachycardia. 

## Discussion

Port-A-Cath® is a propriety central venous access port device (CVAPD) that allows for repeated delivery of drugs, fluids, and nutrition. It is commonly indicated in cancer patients for intermittent delivery of chemotherapy agents over a period of months. The insertion technique employed on our patient involved obtaining venous access via a right internal jugular vein, and the catheter tip was positioned in the superior vena cava. The patient presented one year later with sepsis, and on transesophageal echocardiography, we found the catheter tip prolapsing through the tricuspid valve as the probable cause of the tricuspid regurgitation.

Bassi, et al. retrospectively analyzed long-term complications in 81 patients with implantable venous access ports, classifying them as mechanical and non-mechanical [1]. Of these 81 patients, 45 patients had a site of access through the right internal jugular vein similar to our patient. Among the complications described, thrombotic occlusion and blood stream infections (0.25 per 1,000 device days for both) were the most common. Mechanical complications arising from the proximity to right heart structures were not reported, probably due to the small sample size; such was seen in our patient.

Chrissoheris, et al., in a single-center retrospective review of 24 patients with central venous catheter-associated endocarditis, reported increased right-sided infective endocarditis when the line tip was located within or near the right atrium [2]. However, isolated regurgitation without vegetations was not reported.

Others have also reported isolated tricuspid valve endocarditis following central venous access insertion, including port placement in the superior vena cava [3]. Aoyagi, et al. reported one such case that required surgical treatment [4]. Our case is unique in presentation since the tricuspid regurgitation was likely a mechanical complication of the catheter, which was amenable to removal.

Mutlak, et al. reported that severe tricuspid regurgitation is often functional and related to annular dilatation secondary to aging, atrial fibrillation, or other causes [5]. However, our patient was young and had no evidence of chamber dilatation or pulmonary hypertension. Furthermore, the echocardiographic finding of the intermittent prolapsing catheter tip makes it the likely cause of the coaptation defect. Such inference is also strengthened by the patient’s presentation of supraventricular tachycardia, which may have occurred due to the mechanical contact between the catheter tip and tricuspid annulus, thereby acting as a focus of reentrant circuit.

With regards to the supraventricular tachycardia, the most common variant described is atrial fibrillation followed by three mechanisms with very similar manifestations: atrioventricular nodal reentrant tachycardia (AVNRT), atrioventricular reciprocating tachycardia, and atrial tachycardia. Such manifestations include rapid onset, a heart rate of 150 - 250 bpm, and regular QRS complexes. Reentrant pathways in close proximity to the tricuspid valve are described for atrial flutter and AVNRT [6].

Electrocardiogram tracing in our patient was consistent with a diagnosis of either common AVNRT, orthodromic AVRT, or junctional tachycardia. This is based on the diagnostic algorithm proposed by Buttà, et al. [7]. Given the anatomic defect of coaptation of the tricuspid valve and a possible annular defect, we are inclined to ascribe it to AVNRT in particular.

The arrhythmogenic effect of indwelling central venous catheters is well reported. Hozdic, et al. studied complications of 108 patients who had a central venous catheter (CVC) insertion and placement for more than seven days via the subclavian vein. Arrhythmia was the most common complication (24 cases; 22%) and was most often supraventricular tachycardia or supraventricular extrasystoles [8].

Caudal displacement of catheters placed in the superior vena cava can result in more serious arrhythmias as well. Ventricular arrhythmias and atrial fibrillation have been reported in patients with a peripherally inserted central catheter (PICC) line, which is a widely used long-term CVC [9-10]. Such arrhythmias are referred to as position-dependent arrhythmias in relation to the PICC line since they can be reproduced by moving upper extremity in a particular manner. Additionally, fracture of the catheter tip with a fragment lodging in right atrium has been reported.

## Conclusions

In conclusion, we report a case of severe tricuspid regurgitation and supraventricular tachycardia in a young patient with a long-term central venous port device. While such central venous catheters have been associated with complications, such as endocarditis and arrhythmias, we report a unique case of valvular insufficiency caused by the tip of a catheter prolapsing through the tricuspid valve. Broad differentials should be considered for complications of central venous catheters in such patients, including rare mechanical complications like valvular insufficiency.
